# Incidence and Patterns of Congenital Heart Disease Among Jordanian Infants, a Cohort Study From a University Tertiary Center

**DOI:** 10.3389/fped.2020.00219

**Published:** 2020-05-05

**Authors:** Wasim Khasawneh, Fakhri Hakim, Omayma Abu Ras, Yara Hejazi, Abdullah Abu-Aqoulah

**Affiliations:** ^1^Department of Pediatrics and Neonatology, Faculty of Medicine, Jordan University of Science and Technology, Irbid, Jordan; ^2^Department of Pediatrics, King Abdullah University Hospital, Ramtha, Jordan

**Keywords:** congenital heart disease, infants, cyanotic, acyanotic, patterns of CHD

## Abstract

**Background:** Data is limited about the incidence of congenital heart disease in Jordan. The goal of this study is to determine the incidence and patterns of congenital heart diseases (CHD) among Jordanian infants evaluated at King Abdullah University Hospital.

**Methods:** A retrospective chart review was conducted for all infants who had an echocardiogram evaluation in the 3-years period July 2016–June 2019. All included infants had a 2-dimentional echocardiogram with a Doppler vascular study performed by the same cardiologist. Infants with a structural congenital heart disease were included in the analysis.

**Results:** A total of 1,028 infants were evaluated at the cardiology department during the study period. Eight hundred and sixty-five had an abnormal echo finding. Two hundred and ninety-eighth were diagnosed with CHD. The incidence of CHD was 25 per 1000 live births. Fifty one percent were premature infants. The majority of cases were mild CHD. Patent ductus arteriosus was the most common acyanotic lesion followed by ventricular septal defect and atrial septal defect with a prevalence of 44, 25, and 25%, respectively. Cyanotic CHD constituted 6% of all CHD. Tetralogy of Fallott was the most common cyanotic CHD. The main indication for referral was hearing a heart murmur during physical examination.

**Conclusion:** Although the incidence of CHD in our cohort was relatively high, the majority of cases were acyanotic mild CHD with favorable prognosis. A wider population-based study is needed to evaluate the incidence and better understand the patterns and distribution of CHD at a national level.

## Introduction

Among all congenital defects, congenital heart diseases (CHD) continue to be the major cause of mortality in pediatric age groups. The incidence of CHD varies between populations according to the published studies conducted in different countries with an incidence ranging from 4 to 50 per 1,000 live births (1). According to the CDC, the incidence of CHD in the US is about 1% or 10 per 1,000 live births (2). A systematic review and meta-analysis report showed the incidence in Asia about 9.3/1,000 live birth (3). The variably reported rates of CHD among published studies are related to the difference in their inclusion criteria. Some studies exclude bicuspid aortic valve, tiny ventricular septal defects and silent patent ductus arteriosus (PDA) from their cohort and this could have a significant effect on the reported rates (1). Although not well-explained by strong evidence, the difference might be also affected by multiple environmental and genetic factors. CHD prevalence in developing countries might be underestimated due to lack of proper healthcare systems and follow up, unavailable detection modalities and limited diagnostic techniques (4). In our region in the Middle East, several hospital-based reports have been published showing a prevalence ranging from 2 to 11% (5, 6) but there has been no documented data at national levels.

CHD are categorized into trivial, moderate and severe lesions or acyanotic vs. cyanotic defects according to the pathophysiology and affected heart structure (1). The acyanotic lesions are mostly included in the milder CHD group and this includes septal cardiac defects like atrial septal defect (ASD), ventricular septal defect (VSD), and atrioventricular canal defects. In addition, left ventricular outflow obstructive lesions like aortic stenosis and coarctation of aorta are other examples of acyanotic CHD with more complexity. Cyanotic CHD include Tetralogy of Fallott, transposition of great arteries, total anomalous pulmonary venous returns, hypoplastic left heart syndrome, truncus arteriosus, and tricuspid atresia (1).

Although VSD has been frequently reported to be the most common CHD across the world (1, 2), the patterns of CHD might vary according to different etiologic factors including genetic background, geographic location, seasonal influence, maternal age, and the presence of CHD among other family members. In Jordan, data is limited about the national incidence and pattern of CHD. However, three papers have been published in the past two decades from local hospital analysis indicating that VSD was the most common CHD accounting for 35–44% of all CHD cases (7–9). In their center at Jordan University, Al Ammouri et al. reported an estimated incidence of 12.3 per 1,000 live births (7).

In countries with limited resources, the delay in diagnosing CHD adds to the already existing high mortality and morbidity rates. Therefore, early identification and timely intervention is key in reducing related morbidity and mortality. In developed countries, early detection and proper treatment have increased the survival rate and decreased mortality from 80 to 20% resulting in an increase in the number of adults surviving with CHD (10).

Our study was conducted to review the current prevalence of CHD and to assess the patterns and distribution among Jordanian infants who were referred for cardiac evaluation at our university-based hospital. We also assessed the medical management of certain CHD and the need for referral to a special cardiac center for the surgical cases.

## Methods

After obtaining the Institutional review board approval at Jordan University of Science and Technology (IRB 427-2019), a retrospective chart review was conducted for all infants who had an echocardiogram done at King Abdullah University Hospital in Irbid, Jordan in the period July 2016–June 2019. Our tertiary referral academic hospital serves more than two million people in the North of Jordan. It is the major hospital with pediatric cardiology department that offers cardiology evaluation and referral services to the majority of population residing in the region. During the study period, we didn't have a full time cardiac surgeon in our institution and so all cases with complex CHD or cases that need surgical intervention were transferred out after the diagnosis is established.

Included in this review are all infants who have been evaluated by our cardiologist in the inpatient setting at the neonatal ICU, well baby nursery, pediatric ICU and pediatric floor, as well as in the outpatient clinic department.

In Northern Jordan, pediatric cardiac evaluation and maternal-fetal medicine services are primarily available at our center. Patients with governmental insurance are delivered and followed at other governmental hospitals. The CHD cases included in this cohort were all delivered and evaluated at our institution. According to the health insurance policy in our country, newborns delivered at our institution continue to follow for general care and/or subspecialty care locally, and they are offered a referral to other centers only if cardiac surgical intervention is required. A few antenatally diagnosed cases of complex CHD were not delivered at our institution and so were not included in our cohort.

Data collected include birth weight, gestational age, birth order, maternal age, family history, antenatal diagnosis, indication for echo, follow up findings, the need for CT angiogram to confirm diagnosis, medical management, associated anomalies, the need for referral, and the outcome of survival vs. death.

Two-dimensional echocardiogram with Doppler vascular assessment was performed in all included patients. To ensure validity, all studies were performed and reviewed by the same cardiologist during the study period. The diagnosis of abnormal heart defect was obtained as documented in the electronic medical records by the cardiologist at the time of evaluation.

Infants with any non-trivial abnormal echocardiogram finding underwent a follow up study by the same cardiologist within one to 2 weeks to confirm the abnormality.

We followed Mitchells definition for CHD (11), and so not all cases of abnormal echocardiogram finding were included as CHD cases. Some abnormal echo findings are not related to a structural heart disease and so don't fit the definition of CHD. Examples include cases of persistent pulmonary hypertension (PPHN) and left or right ventricular hypertrophy (LVH/RVH).

Regarding cases of patent ductus arteriosus (PDA), all hemodynamically significant lesions were counted in the total number of CHD. On the other hand, and since some infants had their initial echo study done within the first few days after birth, and to avoid over diagnosing PDA, non-hemodynamically significant PDA lesions were counted only if they were persistent in the follow-up echo study after the age of 1 week. Infants with an isolated patent foramen ovale (PFO) were also excluded from the CHD count.

Data were entered into an excel spreadsheet. Statistical analyses were performed using IBM SPSS Statistics Software (version 23). Data were presented as frequency distributions for categorical variables and mean ± standard error of the mean for continuous variables.

## Results

During the study period, a total of 1,028 infants were referred for cardiology evaluation and each had a 2-dimentional echocardiogram performed at least once. All cases, but one, were referred before the age of 30 days, and more than half had their first study done during the 1st week of life. Eight hundred and sixty-five of the evaluated patients (84%) had an abnormal initial echo finding. After excluding early PDA cases that disappeared on subsequent echo study, isolated PFO cases, and cases of abnormal echocardiogram finding without a structural heart abnormality such as persistent pulmonary hypertension (PPHN) and LVH/ RVH, the number of infants labeled as having a structural CHD was 298 representing an incidence of 2.5% among all live births during the study period (i.e., 25 cases per 1,000 live births). Of the CHD cases, 51% were males and 49% were preterm (Table 1).

**Table 1:** Sociodemographic characteristics of infants with CHD.

**Characteristic**	**Number (*N* = 298)**	**%**
**Gender**		
Male	153	51
Female	145	49
GA, mean (SD)	35 (3.7)	
≥37	151	51
<37	147	49
Weight (gm), mean (SD)	2,612 (866)	
≥2,500	171	57
1,500–2,500	73	25
<1,500	54	18
**Number of Gestation**		
Singleton	245	82
Multiples	53	18
Maternal age (year), mean (SD)	32 (5.5)	
<20	10	3
20–35	211	71
>35	77	26
**Birth Order**		
1	107	36
>1	191	64
DOL echo, mean (SD)	8 (7)	
1	25	8
2–3	53	18
3–30	219	73
>30	1	<1

*CHD, Congenital heart disease; SD, Standard deviation*.

*GA, Gestational age; DOL, Day of life*.

Among all CHD cases, PDA was the most common diagnosis accounting for 43% followed by VSD and ASD with a rate of 25% in each category. Isolated PDA was seen in 31%. The incidence of PDA among <32-weeks premature infants delivered during the study period was 74% (Table 2).

**Table 2:** Distribution of CHD.

**Type of CHD**	**Number**	**% of total CHD**
**Acyanotic**	281	94
Patent ductus arteriosus	132	44
Isolated patent ductus arteriosus	93	31
Ventricular septal defect	75	25
Atrial septal defect	76	25
Atrio-ventricular canal	6	2
Coarctation of aorta	7	2.4
Aortic stenosis	0	0
Peripheral pulmonic stenosis	2	<1
Pulmonary valve stenosis	1	<1
**Cyanotic**	17	6
Tetralogy of Fallott	8	2.7
Transposition of great arteries	5	1.7
Hypoplastic left heart syndrome	1	<1
Truncus Arteriosus	2	<1
Tricuspid Atresia	0	0
Total anomalous pulmonary venous return	1	<1

*CHD, Congenital heart disease*.

Cyanotic CHD constituted 6% of all CHD with an incidence of 1.5 per 1,000 live births during the study period. Tetralogy of Fallot (TOF) was the most common cyanotic CHD followed by transposition of great arteries (TGA).

Hearing a heart murmur during regular physical exam was the most common indication for cardiology referral and was ascertained in 292 cases. Of these cases with murmur, 251 (86%) had an abnormal initial echocardiogram finding. On the other hand, out of the 298 CHD cases, heart murmur was heard in 91. In addition, persistent cyanosis and high oxygen requirement, not exclusively explained by primary lung pathology, was the indication for referral in 165 evaluated cases (Table 3).

**Table 3:** Indication for cardiology referral.

**Indication**	**Among CHD *n* (%)**	**Among all referrals *n* (%)**
Murmur	91 (31)	292 (28)
Cyanosis/ high FiO2	80 (27)	165 (16)
Antenatal suspicion	18 (6)	43 (4)
Family history	8 (3)	21 (2)
Screening	70 (23)	88 (9)
Unknown	31 (10)	417 (41)

*CHD, Congenital heart disease*.

*FiO_2_, Fractionated inspired Oxygen*.

Of all cases, 43 had an antenatal suspicion for CHD, of whom 36 cases were confirmed postnatally. The most common antenatal diagnosis was VSD and the most accurate antenatally suspected diagnosis, as confirmed postnatally, was TOF.

PPHN diagnosis was established in 25 patients (8.4%) of whom 19 cases completely resolved in subsequent echo after receiving the appropriate therapy.

Although 21 infants were initially referred for cardiac evaluation as a screening measure due to the presence of serious CHD among one of their family members, only eight of our CHD cases have at least one other first-degree relative diagnosed with CHD as charted in their electronic medical records.

Due to uncertain diagnosis by echocardiogram in some cases, CT angiogram was performed in four cases. Of those, two were confirmed abnormal; one had a diagnosis of Coarctation of aorta, while the other diagnosed as Truncus arteriosus.

Medical management for hemodynamically significant PDA was commenced in a total of 63 babies (nearly half of all PDA cases) using oral Ibuprofen, Intravenous Ibuprofen or oral Paracetamol in 35, 12, and 16 cases respectively, with a success rate of 80, 82, and 75% as documented with follow up echocardiogram after completion of the treatment course.

Of all cases diagnosed with CHD, 38 (12.7%) had associated anomalies involving other systems, and trisomy 21 was the most commonly established chromosomal abnormality.

The case fatality rate among our CHD cases was 8.7%. Of those babies who died, 46% (12/26) were preterm and 27% (7/26) had complex CHD.

## Discussion

Congenital heart diseases represent a significant global health burden and continue to be the major cause of mortality in infants with congenital birth defects (2). In this retrospective cohort, we reviewed the incidence and patterns of CHD among Jordanian infants at our tertiary academic hospital in North of Jordan.

There seems to be a big variation in the incidence of CHD between different nations. This variation might be affected by the inclusion criteria of each particular study such as the age of included cases, the size of certain heart defects and the methodology applied for diagnosis (1, 12). The incidence of all CHD among our studied population is 2.5%. In Jordan, there have been no previous studies focusing on the national incidence of CHD to use as a reference and to compare our findings with. In a hospital based retrospective review, Al Ammouri reported an estimated incidence of 12.3 per 1,000 live births from Jordan University hospital which is a major academic referral institution in the Jordanian capital city of Amman (7). The reported rates have been <1% in Europe and USA (2, 13). In the Arab world and other developing countries, the incidence of CHD is higher than the Western countries. For example, Saidan et al. reported a prevalence of nearly 1.5% in their most recently published study from Saudi Arabia (6). In India, several papers were published reporting a CHD incidence of 4-26 per 1,000 live births (14). Although the difference in the prevalence between different populations is not well-studied and not well-explained by strong evidence, this variation might be related to multiple genetic and environmental factors including ethnicity and consanguineous marriage (6, 15).

According to CDC, the prevalence of certain CHD like PDA and septal heart lesions is increasing, while the prevalence of some other CHD is decreasing (2). The increased rate of CHD could be also explained by the fact that more cases are discovered antenatally to have suspected CHD, and so they get early referral for cardiac evaluation. Antenatal detection helps in avoiding delayed diagnosis and minimizing the related consequences (1, 16). In addition, antenatal diagnosis is expected to improve the operative outcome and potentially the overall prognosis by having the mothers deliver at tertiary care centers with special pediatric cardiac units. This also allows parental counseling and preparation to the expected outcome (16). In our cohort, of the 43 cases with suspected CHD by antenatal screening, only 40% were confirmed by postnatal echo.

Another major factor that could have affected the rate of CHD diagnosis is the implementation of critical CHD pulse oximetry screening tool in the newborns. With this screening, the chance of missing CHD cases has declined and the outcome of diagnosed cases has improved (17, 18).

The pattern of distribution of CHD in our study was somehow different from other reports worldwide. PDA was the most common CHD among our studied population followed by VSD and ASD. Most worldwide CHD studies have reported VSD to be the most common CHD (1, 6). The high PDA prevalence in our cohort could be explained by the fact that 70% of our PDA diagnosed infants were premature. High rate of PDA has been also reported in Pakistan (19) and India (20). Some studies excluded PDA from CHD unless they are hemodynamically significant while others included PDA only if it persisted beyond certain age, and this explains the big variation in the reported rate of PDA.

Our relatively higher incidence of CHD could be explained by the fact that all our included cases were evaluated and labeled as CHD in the 1st month of life. Since PDA, VSD and ASD account for more than 90% of these cases, and since these lesions have a chance of a spontaneous closure without causing any significant impact on health and without requiring any medical intervention, the incidence of CHD in our cohort could have been lower if we had reassessed all patients at older age during childhood or adolescence (21).

Most data about CHD from developing countries are hospital-based retrospective reviews with few reports published about the status at national levels. In Saudi Arabia, the prevalence of CHD was reported to be around 15 per 1,000 live birth. In their prospective report from a local Saudi district, ASD was the most commonly diagnosed lesion, while complex CHD accounted for around 20% (6). In Palestine, a review of CHD at four local cardiac centers in Ghaza strip reported a CHD incidence of 10 per 1,000 live births with VSD being the most commonly diagnosed lesion (22). Rahim et al. reviewed the CHD patterns in a local province in Iran and concluded a total incidence of 12.3 per 1,000 with ASD and TOF to be the most commonly diagnosed lesions (23).

The outcome of our PDA diagnosed cases was great. The availability of pharmacological therapy for hemodynamically significant PDA had contributed to the improved outcome of premature babies with this defect resulting in a smaller number of babies with persistent symptoms of congestive heart failure and less need for surgical intervention. The success rate among <32-weeks premature infants in our study using enteral or Intravenous Ibuprofen or enteral Paracetamol was around 75% with comparable results among all three choices. This rate of success has been consistent, and even better, when compared with other studies (21, 24).

The rate of cyanotic lesions among CHD cases in our study was 6%. This rate is quite low when compared with other worldwide studies (1). In the their study from Jordan university, Al Ammouri reported that 11% of their cohort to have cyanotic CHD (7). In both studies, TOF was the most common cyanotic CHD and this finding is consistent with other studies (1). The low rate of cyanotic CHD among our cohort could be explained by the fact that some antenatally diagnosed cases were transferred before delivery to other hospitals in the capital city of Amman where cardiac surgery is more readily available.

Hearing a heart murmur was the most common indication for referral to the cardiology department. Eighty-six percent of infants with a heart murmur had an abnormal initial echocardiogram study. This signifies the importance of thorough physical examination to all infants in the inpatient and outpatient settings.

CHD is seen as an isolated birth defect or in association with other systemic anomalies. The prevalence of coexisting anomalies in our study was 13% compared with 40% in KSA and around 25% in Europe and India (13, 25). Trisomy 21 was the most common chromosomal abnormality detected in babies with combined CHD and other systemic anomalies. Trevisan from Brazil reported that Trisomy 21 accounted for nearly 40% of chromosomal abnormalities in patients with CHD (26).

CHD continue to be listed as the commonest cause of mortality in infants with congenital anomaly (1). According to CDC, nearly half the deaths due to CHD occur during infancy. The rate of mortality among our CHD cases was 8.7%, of whom 46% (12/26) were preterm, and 27% (7/26) had complex CHD. The mortality rate from Saudi Arabia was reported to be around 17% (6).

Our study is not without limitations. The major limitation is the retrospective nature of this cohort; our data relied completely on the electronic medical record documentation. However, having all cases evaluated by the same cardiologist could be considered a point of strength to avoid inter-operator bias. Also, being a single center study makes it difficult to infer conclusions about the true prevalence or incidence of CHD at a national level. Another limitation is related to the unexpectedly low antenatal detection rate which might be explained by the fact that a good percentage of pregnant women might miss the 20-weeks anatomy scan as they receive early antenatal care with private practitioners and start to follow at our institution toward the end of gestation. In addition, cases of complex CHD that got referred outside our institution prior to delivery were not included in our cohort and this could have impacted the incidence and patterns of certain complex CHD.

In conclusion, this single center retrospective cohort reports a high incidence of CHD among our hospital-based Jordanian infants compared with the Western countries and other nations in the region. The patterns of CHD were also different, but the overall outcome was excellent. Most CHD cases are benign acyanotic lesions belonging to the mild category of CHD. The rate of coexisting anomalies and case fatality rate is low compared with others. Further prospective studies involving multiple hospitals with surgical centers in particular are needed to better evaluate the incidence and patterns of CHD at a national level. The absence of a national database about CHD in Jordan should raise an alarm about this serious congenital anomaly, and should encourage all decision makers to meet in order to establish and implement national guidelines to monitor this serious health burden.

## Data Availability Statement

All data collected is available from the corresponding author upon reasonable request.

## Ethics Statement

The studies involving human participants were reviewed and approved by IRB at Jordan University of Science and Technology. IRP number 427-2019. Written informed consent for participation was not required for this study in accordance with the national legislation and the institutional requirements.

## Author Contributions

WK substantially contributed to the study design, created the study data collection sheet, reviewed and edited all the collected data, participated in data analysis, drafted and reviewed the manuscript. FH reviewed and edited all the collected data, drafted and reviewed the manuscript. OA and YH contributed to data collection, data analysis and manuscript revision. AA-A contributed to study design, data collection, and literature review. All authors gave final approval of the version to be published and agreed to be accountable for its contents.

## Conflict of Interest

The authors declare that the research was conducted in the absence of any commercial or financial relationships that could be construed as a potential conflict of interest.
